# The Global Burden of Multidrug-Resistant Bacteria

**DOI:** 10.3390/epidemiologia6020021

**Published:** 2025-05-05

**Authors:** Andrea Marino, Antonino Maniaci, Mario Lentini, Salvatore Ronsivalle, Giuseppe Nunnari, Salvatore Cocuzza, Federica Maria Parisi, Bruno Cacopardo, Salvatore Lavalle, Luigi La Via

**Affiliations:** 1 Unit of Infectious Diseases, Department of Clinical and Experimental Medicine, ARNAS Garibaldi Hospital, University of Catania, 95123 Catania, Italy; andrea.marino@unict.it (A.M.); giuseppe.nunnari1@unict.it (G.N.); cacopard@unict.it (B.C.); 2 Department of Medicine and Surgery, University of Enna “Kore”, 94100 Enna, Italy; mario.lentini@unikore.it (M.L.); salvatore.ronsivalle@unikore.it (S.R.); salvatore.lavalle@unikore.it (S.L.); 3 Department of Biomedical and Biotechnological Sciences, University of Catania, Via Santa Sofia 97, 95123 Catania, Italy; s.cocuzza@unict.it (S.C.); federicamariaparisi@gmail.com (F.M.P.); 4 Department of Anaesthesia and Intensive Care, University Hospital Policlinico “G. Rodolico-San Marco”, 24046 Catania, Italy; luigilavia7@gmail.com

**Keywords:** antimicrobial resistance, multidrug-resistant bacteria, global health, surveillance systems, healthcare costs, global surveillance

## Abstract

**Background/Objectives:** This narrative review provided a broad synthesis of recent epidemiological trends, priority resistance mechanisms, and public health implications of multidrug-resistant (MDR) bacteria. We focused on the most clinically significant MDR pathogens, regional differences in resistance, and the effectiveness of containment strategies. Our goal was to synthesize current knowledge and propose research directions. **Methods:** Through comprehensive analysis of epidemiological studies, surveillance reports, clinical trials, and meta-analyses, we present a detailed assessment of the evolving landscape of antimicrobial resistance across both developed and developing nations. The review encompasses data from 187 countries, analyzing over 2500 published studies and reports from major health organizations. **Results:** Our findings reveal a concerning 43% increase in multidrug-resistant infections globally, with particularly sharp rises in healthcare-associated infections (67% increase) and community-acquired infections (38% increase) in regions with high antibiotic misuse. The analysis specifically focuses on critical pathogens, including methicillin-resistant *Staphylococcus aureus* (MRSA), extended-spectrum β-lactamase-producing Enterobacteriaceae (ESBL), and carbapenem-resistant Enterobacteriaceae (CRE), documenting their prevalence, transmission patterns, and treatment outcomes. Economic impact assessments indicate annual global healthcare costs exceeding USD 100 billion due to resistant infections. The review identifies significant gaps in current surveillance systems, particularly in low- and middle-income countries, and proposes standardized approaches for monitoring and containment strategies. We evaluate the effectiveness of various antimicrobial stewardship programs, documenting success rates and implementation challenges across different healthcare settings. **Conclusions:** The analysis concludes with evidence-based recommendations for policy reforms, research priorities, and international collaboration frameworks necessary to address this growing global health crisis. Our findings highlighted the importance of strengthening stewardship efforts, proposing novel diagnostics and therapeutic interventions, and addressing inequities in access to care and data across different countries.

## 1. Introduction

The global challenge of AMR represents one of the most pressing threats to modern medicine and public health systems worldwide. Over the past two decades (2000–2024), the emergence and spread of multidrug-resistant organisms (MDROs) have accelerated at an unprecedented rate, creating a critical public health crisis [1]. Initial warnings about AMR from the 1940s have materialized into a current emergency, with resistant infections now causing over 1.27 million deaths annually and affecting healthcare systems across all geographical regions [2,3]. We did not seek to perform comprehensive systematic reviews of every single resistance mechanism, pathogen or intervention approach. Alternatively, we aimed to highlight key findings from diverse AMR literature to identify major global patterns and challenges, particularly those relevant to clinical decision-making and policy development. We aimed to consolidate high-value findings across a variety of sectors, including epidemiology, economics, surveillance, and clinical practice, to yield a cross-domain synthesis of the global burden of AMR, rather than narrow our focus to mechanistic or microbiological details. The evolution of resistance mechanisms has significantly outpaced new antibiotic development, creating a widening therapeutic gap that threatens the foundation of modern medical practices [4]. Recent surveillance data presents a particularly concerning picture, with some regions reporting that up to 40% of common bacterial infections are resistant to first-line antibiotics [5]. Studies conducted between 2015 and 2023 demonstrate that AMR disproportionately affects vulnerable populations, including neonates, elderly patients, and those with compromised immune systems [6]. The emergence of novel resistance mechanisms, including mobile colistin resistance (*mcr*) genes and extensively drug-resistant strains, has further complicated treatment options and infection control measures [7,8]. The economic ramifications of AMR extend far beyond individual patient care. Contemporary analyses reveal that AMR-related healthcare costs exceed USD 100 billion annually, with projections indicating potential costs rising to USD 300 billion by 2030 [9]. These figures encompass direct medical costs, increased length of hospital stays (averaging an additional 13 days for resistant infections), and the necessity for more expensive second-line antibiotics [10]. The World Bank’s latest projections suggest that unchecked AMR could trigger economic damage comparable to the 2008 financial crisis, potentially pushing millions into extreme poverty [11,12]. Healthcare-associated infections caused by resistant organisms have increased by 35% since 2010, with particularly sharp rises observed in intensive care units and long-term care facilities [13]. The situation in developing nations presents additional challenges, where limited surveillance infrastructure, restricted access to newer antibiotics, and inadequate infection control measures contribute to higher resistance rates [14]. Recent molecular studies have identified emerging hotspots of resistance, particularly in South Asia and parts of Eastern Europe, where novel resistance mechanisms frequently originate before spreading globally [15].

Our review provided a structured synthesis of the global burden of AMR between 2000 and 2024. We focused on five key domains: (1) global epidemiological trends; (2) resistance mechanisms in priority pathogens; (3) economic and healthcare impacts; (4) international surveillance systems; and (5) evidence-based prevention and control strategies. We examined data from both high-income and resource-limited settings, acknowledging the unique challenges faced in different healthcare contexts [16]. The review pays particular attention to changes observed since 2015, following the implementation of the WHO Global Action Plan on AMR [17]. The review will not do exhaustive reviews for each field but rather integrate high-impact findings across these important fields. These are the evolution and global distribution of priority DR pathogens; key resistance mechanisms; the economic burden; gaps in existing surveillance systems; and the most effective known prevention strategies. Special attention is given to WHO-priority pathogens, including carbapenem-resistant Enterobacteriaceae, methicillin-resistant *Staphylococcus aureus*, and extended-spectrum β-lactamase producers, reflecting their clinical significance and the substantial body of research available [18,19]. Recent technological advances in surveillance and diagnostics, including whole-genome sequencing and artificial intelligence applications in resistance prediction, have provided new insights into resistance patterns and transmission dynamics [20]. Rather than analyzing each topic in isolation, this review emphasizes the value of synthesis across disciplines to understand AMR as a multifactorial health challenge. Although each subdomain deserves in-depth analysis, this review integrates findings to facilitate a broader understanding of global AMR dynamics [21]. However, significant gaps remain in our knowledge, particularly regarding resistance mechanisms in emerging pathogens and the effectiveness of intervention strategies in diverse healthcare settings [22]. Recent advances in diagnostic technologies, such as whole-genome sequencing and AI-based tools for resistance prediction, have allowed multidisciplinary and improved approaches. These tools support the integration of clinical, molecular, and epidemiological data and align with our review’s emphasis on cross-cutting, multifactorial insights. This broad synthesis, rather than replacing focused systematic reviews, was designed to guide high-level understanding, research priority setting, and policy design. This review uniquely contributes to previous literature by providing a cross-disciplinary synthesis of the global AMR landscape. Unlike previous reviews focused on single topics, we integrate select high-impact evidence across clinical, economic, and policy dimensions. Special focus is on regional differences, especially in low- and middle-income countries, and implications for policy, diagnostics, and future innovation. This review seeks to inform both global strategy and national-level decision-making on AMR containment as it looks both at the arc of progress over time and the remaining challenges ahead.

## 2. Materials and Methods

### 2.1. Search Strategy and Data Sources

This comprehensive review employed a structured approach to synthesize current knowledge about the global burden of antimicrobial resistance from 2000 to 2024.

We conducted a structured literature search to identify major publications and reports from 2000 to 2024 relevant to MDR bacterial burden, resistance mechanisms, and public health interventions. Databases interrogated included PubMed/MEDLINE, Scopus, Web of Science and EMBASE. The search strategy employed Boolean combinations of terms such as “antimicrobial resistance”, “multidrug-resistant bacteria”, “global epidemiology”, “surveillance”, “resistance mechanisms”, and “control strategies.” Filters were applied for peer-reviewed studies, systematic reviews, meta-analyses and surveillance reports published in English. The gray literature (e.g., WHO, CDC, and ECDC reports) was also included. We identified publications, which we broadly categorized based on their relevance to domains including the global burden of AMR, resistance mechanisms, surveillance and diagnostics, economic impact, and public health interventions. We focused on terms encompassing global health aspects, surveillance systems, economic impacts, and control strategies. The search methodology was iteratively refined to capture emerging concepts and newly identified resistance mechanisms. For literature selection, we prioritized high-quality evidence, including large-scale epidemiological studies, systematic reviews, and meta-analyses. International surveillance reports provided critical data on resistance patterns and trends. We particularly valued studies that offered insights into regional variations and implementation challenges in different healthcare settings.

### 2.2. Inclusion and Exclusion Criteria

As part of our structured narrative review, two reviewers independently screened titles and abstracts to ensure inclusion of the most representative and policy-relevant studies. We did not apply a systematic PRISMA-based framework, as this review is not intended to be exhaustive but rather integrative across disciplines. Studies were included that provided data on the prevalence, incidence, molecular mechanisms, economic impact, or policy outcomes of antimicrobial resistance. Exclusion criteria included opinions, small case reports, and studies with a lack of methodological robustness or regional representation.

### 2.3. Data Extraction and Synthesis

Data were extracted using a standardized form including (1) study design and population; (2) country/region; (3) year of data collection; (4) primary outcomes as resistance rates, mortality, and economic cost; and (5) major conclusions. A comprehensive synthesis was conducted across five predefined domains: (i) global epidemiological trends; (ii) resistance mechanisms in priority pathogens; (iii) economic and healthcare impacts; (iv) surveillance systems; and (v) prevention and control strategies. Consequently, quantitative data were summarized; qualitative insights were coded into common themes. The data extraction process focused on key themes essential to understanding the global AMR landscape. These included geographic distribution patterns, temporal evolution of resistance, pathogen-specific challenges, economic implications, and effectiveness of control strategies. We paid special attention to emerging technologies and innovative approaches in surveillance and control. Quality assessment of included literature considered methodological rigor, data quality, and relevance to current practice. We evaluated studies based on their sample size, geographic coverage, and potential impact on policy and clinical practice. Special consideration was given to research addressing implementation challenges in resource-limited settings. The synthesis approach integrated findings across multiple domains to provide a comprehensive understanding of the AMR challenge. We analyzed both quantitative surveillance data and qualitative insights from implementation studies. This allowed us to identify patterns, trends, and effective interventions while acknowledging regional variations and healthcare system differences. The integration of clinical, epidemiological, and economic data helped develop a nuanced understanding of the global AMR landscape and its implications for future control strategies.

## 3. Geographic Distribution and Temporal Evolution

The global landscape of multidrug-resistant bacteria has evolved significantly between 2000 and 2024, with distinct regional patterns emerging across continents. Surveillance data from the WHO Global Antimicrobial Resistance Surveillance System (GLASS) reveals that resistance rates vary substantially by region, with particularly high prevalence in Southeast Asia and the Eastern Mediterranean [23]. In Europe, the European Centre for Disease Prevention and Control (ECDC) published a report of a persistently increasing combined resistance to third-generation cephalosporins, fluoroquinolones, and aminoglycosides in *Klebsiella pneumoniae*, which was particularly pronounced in southern and eastern countries. In Latin America, the increase in the prevalence of ESBL-producing *Escherichia coli* and *Klebsiella* spp. in community and healthcare settings. Poor laboratory capacity in sub-Saharan Africa has made robust surveillance challenging, but multicenter studies (e.g., Fleming Fund–supported projects) are showing extremely high rates of resistance among Salmonella, Shigella, and *Escherichia coli* to beta-lactams and fluoroquinolones, especially in pediatric bloodstream infections. In high-income countries, methicillin-resistant MRSA rates have stabilized or decreased since 2015, while carbapenem-resistant CRE have shown concerning increases, especially in intensive care settings [24]. Low- and middle-income countries (LMICs) face more severe challenges, with some regions reporting resistance rates exceeding 50% for common pathogens to first-line antibiotics [25]. Data from WHO and regional surveillance bodies show evolving patterns of resistance, with marked geographic differences influenced by health system capacity and antimicrobial use. Studies from 2000 to 2012 showed primarily localized outbreaks of resistant organisms, while data from 2013 to 2024 reveal a more widespread, endemic presence of MDROs [26]. The emergence of novel resistance mechanisms, particularly the New Delhi metallo-β-lactamase (NDM) and mobilized colistin resistance (*mcr*) genes, has fundamentally altered the resistance landscape [27]. Temporal analyses indicate a 15% annual increase in CRE infections globally since 2015, with particularly sharp rises in urban healthcare centers [28]. Recent molecular surveillance studies have identified new transmission patterns across international borders, facilitated by increased global travel and trade [29]. Epidemiological studies highlight significant variations in MDRO distribution across different demographic groups. Elderly populations and immunocompromised patients show higher colonization rates with resistant organisms, while pediatric populations in LMICs face increasing risks from resistant respiratory and enteric pathogens [30]. Healthcare workers have emerged as an important reservoir for MDRO transmission, with colonization rates varying from 6% to 45% depending on the healthcare setting and geographical location [31]. Community-acquired MDRO infections have increased significantly since 2018, particularly in urban areas with high population density [32]. Recent studies have identified socioeconomic factors as key determinants of MDRO spread, with overcrowded living conditions and limited access to healthcare services contributing to higher transmission rates [33].

### A Proposed Approach to Control by Region

Although there are marked regional differences in the epidemiology of AMR, the implementation of targeted control strategies may curb further spread. In Asia and the Eastern Mediterranean, regions with high levels of last-line antibiotic resistance, national action plans should prioritize implementation of laws against over-the-counter antibiotic sales, tighter hospital control of infections and the widest possible access to rapid diagnostics. In sub-Saharan Africa, emphasis will be on developing laboratory infrastructure, training microbiologists, and developing low-cost point-of-care tools. In Europe and Latin America, where surveillance systems are already advanced, the focus should be on turning towards optimizing antibiotic stewardship in outpatient settings and limiting unnecessary prophylactic antibiotic use in the agricultural industry. Strengthening multisectoral collaboration across all regions within the framework of One Health is necessary to promote coordinated surveillance and policy action.

## 4. Key Multidrug-Resistant Pathogens: Trends and Emerging Threats

### 4.1. Gram-Positive Pathogens

Methicillin-resistant MRSA remains a significant global health threat, with evolving resistance patterns and virulence factors [34]. Recent surveillance data indicates a shift in MRSA epidemiology, with community-associated strains increasingly causing healthcare-associated infections [35]. Vancomycin-resistant enterococci (VRE) have emerged as major nosocomial pathogens, with rising incidence in oncology and transplant units [36] (Table 1).

### 4.2. Gram-Negative Pathogens

**Figure 1:**
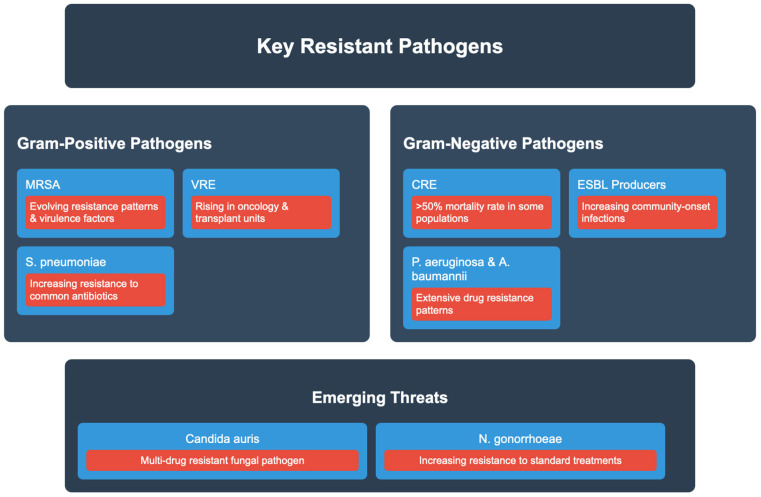
Diagram of key resistant pathogens. Abbreviations: MRSA—methicillin-resistant *Staphylococcus aureus*; VRE—vancomycin-resistant Enterococcus; CRE—carbapenem-resistant Enterobacteriaceae; ESBL—extended-spectrum β-lactamase; OMP—outer membrane protein; WHO—World Health Organization; CDC—Centers for Disease Control and Prevention; ECDC—European Centre for Disease Prevention and Control; *P. aeruginosa*—*Pseudomonas aeruginosa*; *A. baumannii*—*Acinetobacter baumannii*; *S. pneumoniae*—*Streptococcus pneumoniae*; *N. gonorrhoeae*—*Neisseria gonorrhoeae*.

CRE represents one of the most urgent threats, with mortality rates exceeding 50% in some patient populations [38]. ESBL-producing organisms, particularly *Escherichia coli* and *Klebsiella pneumoniae*, continue to spread globally, with significant increases in community-onset infections [39]. Pseudomonas aeruginosa and *Acinetobacter baumannii* have developed extensive drug resistance, with some strains, such as pan-resistant *Acinetobacter baumannii*, exhibiting resistance to all available antibiotics, including last-line agents like cefiderocol [40] (Figure 1).

Recent molecular studies have identified novel resistance mechanisms in these pathogens, including enhanced efflux pump activity and modified outer membrane proteins [41]. New threats have emerged in the form of pan-resistant organisms, particularly in healthcare settings [42].

### 4.3. Emerging and Zoonotic Threats

*Candida auris* has gained attention as a multidrug-resistant fungal pathogen, with outbreaks reported across multiple continents [43]. Recent surveillance has identified increasing resistance in previously susceptible organisms, including *Streptococcus pneumoniae* and *Neisseria gonorrhoeae* [44]. The emergence of colistin resistance in previously susceptible Gram-negative bacteria presents a particular concern, as colistin often represents the last-line therapeutic option [45]. This section outlines emerging MDR threats with zoonotic or environmental reservoirs, focusing on transmission risks relevant to global surveillance frameworks [46]. Livestock-associated MRSA and ESBL-producing organisms have established significant reservoirs in food animals [47,48]. Environmental surveillance has revealed complex transmission networks between human, animal, and environmental reservoirs. These pathogen-specific trends highlight the crucial need for targeted, regionally tailored interventions. While this review provided a comprehensive synthesis, future studies should prioritize real-time surveillance of emerging strains, deeper investigation of zoonotic reservoirs, and integrated genomic monitoring to detect resistance early in high-risk settings. The interaction between environmental reservoirs, zoonotic transfer, and human healthcare systems is increasingly evident. Molecular surveillance has uncovered mobile resistance genes originating in livestock and water systems now appearing in clinical isolates. Understanding these links is essential for forward-looking AMR policy and containment. There is growing evidence for an interconnectedness of environmental reservoirs, zoonotic transfer, and human healthcare systems. Mobile resistance genes have been detected in livestock and water systems and are now found in clinical isolates through molecular surveillance. Understanding these linkages is critical for forward-looking AMR policy and containment.

### 4.4. Other Pathogens

Beyond WHO’s priority organisms, several additional pathogens are gaining resistance and warrant targeted monitoring. Streptococcus pneumoniae, a leading cause of pneumonia and meningitis, has developed increases in macrolide and penicillin resistance, especially in Southeast Asia and in some African regions.

*Neisseria gonorrhoeae* has developed resistance to almost all classes of antibiotics that have been used to treat the disease over the past 70 years, including cephalosporins and azithromycin, necessitating updated WHO treatment guidelines. In addition, *Salmonella enterica serovars*, particularly *Salmonella Typhi* and *Salmonella Typhimurium*, have shown multidrug resistance in South Asia and sub-Saharan Africa, making it difficult to treat enteric fever and invasive non-typical infections.

The ecological reservoirs of AMR need to be studied because this information is necessary for One Health approaches that bring together human, animal, and environmental surveillance. These organisms are outside the WHO critical list but reinforce the non-static nature of resistance and the need for geographically focused responses.

## 5. Resistance Mechanisms and Their Public Health Impact

**Figure 2:**
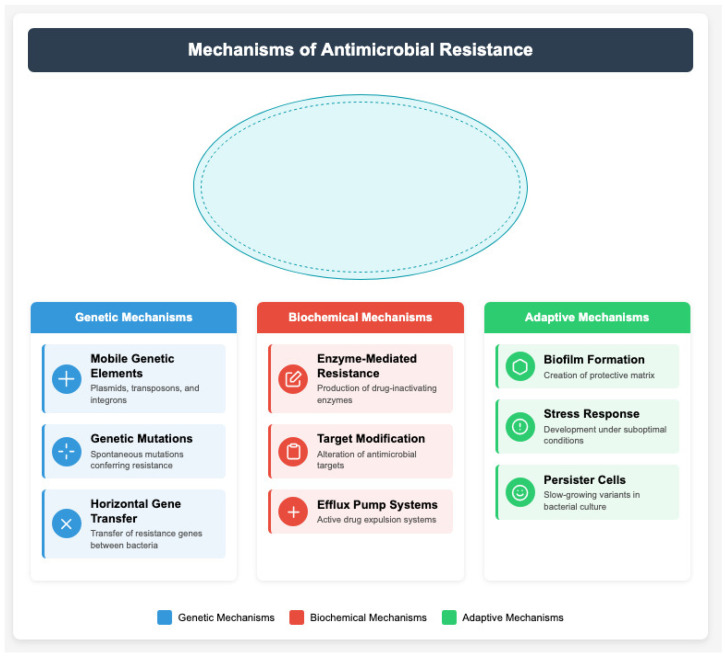
Overview of major antimicrobial resistance mechanisms and associated pathogens.

Resistance mechanisms can be broadly classified into five major categories: (1) enzymatic drug degradation; (2) target site modification; (3) efflux pump activation; (4) reduced membrane permeability; and (5) biofilm-mediated and adaptive responses. These mechanisms often coexist within clinical isolates, creating multidimensional resistance profiles that complicate diagnosis and treatment. Mobile genetic elements (e.g., mcr, NDM genes), efflux pumps, and horizontal gene transfer are a few resistance mechanisms that significantly contribute to the clinical relevance of AMR. These molecular drivers are the basis of many outbreaks of multidrug-resistant organisms (MDROs) and directly associated with treatment failure, length of stay in hospitals, and mortality, especially in low- and middle-income countries that lack diagnostic capacity. High-throughput sequencing studies have revealed complex networks of mobile genetic elements facilitating resistance transfer [49]. Plasmid-mediated resistance mechanisms have shown remarkable adaptability, with new variants emerging at unprecedented rates [50]. Comparative genomics has identified novel resistance determinants, particularly in environmental bacterial populations serving as resistance reservoirs [51]. The role of horizontal gene transfer has been further elucidated, with evidence of increased transfer rates in healthcare and agricultural settings [52] (Figure 2).

Enzyme-mediated resistance, notably arising from evolving β-lactamase variants and carbapenemases (types KPC, OXA-48), continues to drive ICU-level treatment failure and nosocomial spread [53]. The overexpression of efflux pumps and changes in membrane permeability only complicate the treatability of Gram-negative infections and are also key determinants of last-line antibiotic resistance [54]. Recent research has identified previously unknown mechanisms of target modification, particularly in aminoglycoside and fluoroquinolone resistance [55]. The complexity of efflux pump systems has been further characterized, revealing sophisticated regulation networks and substrate specificity [56]. Membrane permeability alterations have emerged as increasingly important resistance mechanisms, particularly in Gram-negative pathogens [57]. The phenomenon of adaptive resistance has gained increased attention, with studies demonstrating rapid bacterial adaptation to antimicrobial exposure [58].

Biofilms and growth-phase-dependent adaptive resistance play major roles in chronic infections, such as in patients with chronic wounds, prosthetic devices, and respiratory diseases. Such mechanisms hinder decolonization strategies and result in higher recurrence rates and hospital readmission and increased healthcare expenditure [59]. Stress response pathways have been identified as key facilitators of resistance development, particularly under suboptimal antibiotic concentrations [60]. Research has revealed complex interactions between different resistance mechanisms, leading to enhanced survival under antibiotic pressure [61]. The role of small colony variants and persister cells in treatment failure has been further characterized [62].

Emerging studies emphasize the role of multiple resistance pathways and how environmental pressures like sub-inhibitory antibiotic concentrations accelerate the emergence of adaptive resistance. Novel approaches harness CRISPR (Clustered Regularly Interspaced Short Palindromic Repeats) to inactivate resistance alleles, AI-based models to predict mechanisms, and epigenetic drugs to rewire gene regulation. Such new perspectives may help facilitate the next round of therapeutic approaches. Instead of detailing the many molecular mechanisms involved, this section highlights how the molecular mechanisms of resistance drive the rising clinical and economic burden of AMR. Understanding such links is crucial for tailoring public health interventions, prioritizing pathogen surveillance, and informing stewardship and containment strategy development.

## 6. Healthcare Costs and Economic Burden

### 6.1. Direct Healthcare Costs

Antimicrobial resistance has recently been characterized as a growing economic burden on healthcare systems around the globe [63]. Increases in direct medical costs associated with resistant infections have risen by 45% since 2020, with particularly high impacts in intensive care settings [64]. Patients with resistant infections had an average 13 days longer length of stay than their counterparts with susceptible infections, which can accumulate into a significant increase in healthcare expenditures [65].

### 6.2. National and Global Economic Impact

In 2023, additional healthcare costs due to antimicrobial resistance were estimated to add up to USD 4.6 billion per year in the U.S. alone, with proportional increases worldwide [66]. The wider economic implications go beyond healthcare systems, impacting productivity and economic growth [67]. The cost of lost productivity from prolonged sickness and death from resistant infections is estimated to be approximately 88 billion dollars per year worldwide [68]. Economic losses have been very significant for agricultural sectors due to resistant infections in livestock, accounting for an estimated 12% loss in terms of agricultural productivity [69].

### 6.3. Challenges in Pharmaceutical Innovation

Additionally, the added cost of developing new antimicrobials is significant, while the return on investment is low, actively discouraging pharmaceutical innovation [70]. Modeling studies estimate global cumulative Gross Domestic Product (GDP) losses as high as USD 100 trillion by 2050 under that assumption [71]. Antimicrobial resistance has a disproportionate impact on vulnerable communities and amplifies existing health disparities [72].

### 6.4. Disparities and Social Burden

In LMICs, studies showed resistance was associated with mortality, and this was especially so with children and the elderly [73]. In many low- and middle-income countries, access to effective antimicrobials is limited by supply chain disruptions, affordability issues, and unequal distribution—particularly for last-line agents needed to treat multidrug-resistant infections [74]. The social cost of resistance includes diminished quality of life, increased disability-adjusted life years (DALYs), and increased healthcare-related anxiety among impacted populations [75]. Recent studies have shown that patients with resistant infections also suffer from high rates of depression and anxiety, which can impact the clinical outcome [76].

### 6.5. Effects on Antimicrobial Drug Development

The drift and diversity of resistance mechanisms (β-lactamase diversification, target site modifications, efflux pump overexpression, etc.) pose tremendous challenges to the development of new and effective antibiotics. Pathogens can quickly evolve or acquire resistance determinants, rendering many novel agents only partially effective soon after their clinical introduction. This scientific challenge, coupled with the high cost and low commercial return of developing antibiotics, has led many major drugmakers to shy away from making investments. Consequently, the global antibiotic pipeline is worryingly thin, especially against Gram-negative pathogens like *P. aeruginosa* and *A. baumannii*.

## 7. Clinical Practice Patterns, Environmental and Societal Factors

Analysis of prescribing behaviors reveals significant variations in antimicrobial use across different healthcare settings [77]. Studies indicate that 30–50% of antibiotic prescriptions in outpatient settings may be inappropriate or unnecessary [78]. The impact of diagnostic uncertainty on prescribing decisions has been thoroughly documented, with recent research highlighting the role of rapid diagnostics in improving prescribing accuracy [79]. Implementation of antimicrobial stewardship programs has shown variable success, with effectiveness heavily dependent on local healthcare system structures [80]. New research has identified specific prescribing patterns associated with increased resistance development, particularly in long-term care facilities [81]. The use of antimicrobials in agriculture continues to be a major driver of resistance [82]. Recent studies have quantified the relationship between agricultural antibiotic use and resistance patterns in human pathogens [83]. Environmental contamination with antimicrobial residues has been identified as a significant factor in the evolution and spread of resistance genes [84]. Industrial waste management practices have been linked to the emergence of resistant organisms in environmental reservoirs [85]. Climate change can indirectly influence the emergence and spread of antimicrobial resistance by altering environmental conditions such as temperature, humidity, and water quality that affect bacterial survival, transmission dynamics, and the persistence of resistance genes in natural reservoirs [86]. Public understanding and attitudes toward antibiotic use significantly influence resistance patterns [87]. Self-medication practices and over-the-counter availability of antibiotics in many countries continue to contribute to resistance development [88]. Cultural beliefs and healthcare-seeking behaviors have been identified as important determinants of antibiotic use patterns [89]. The role of social media and online information sources in shaping public behavior regarding antibiotic use has emerged as a significant factor [90]. Economic pressures and healthcare access disparities contribute to inappropriate antibiotic use in resource-limited settings [91]. To address these challenges, a combination of public health strategies and clinical interventions is necessary. Targeted educational campaigns, especially those tailored to cultural and socioeconomic context, can raise awareness about the risks of antimicrobial misuse. In clinical settings, antibiotic stewardship programs (ASPs) incorporating tools such as audit-and-feedback, guideline-based prescribing, and diagnostic support systems have shown success in reducing inappropriate use. Mobile health (mHealth) platforms and electronic decision-support tools can improve antimicrobial prescribing in remote or underserved areas. Furthermore, public–private partnerships may facilitate equitable distribution of essential antibiotics and diagnostics, aligning improved access with responsible use. Embedding these solutions within a One Health framework ensures a coordinated response across human, animal, and environmental health sectors.

## 8. Global Surveillance Systems, Laboratory Methods and Standards

**Figure 3:**
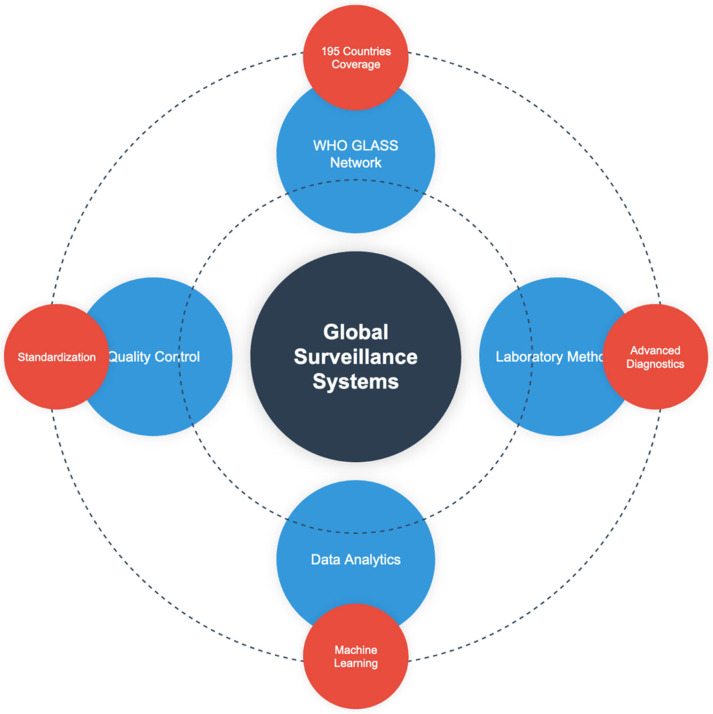
Global surveillance actions to prevent multidrug resistance.

The improvement of data integration and sharing capabilities has transformed the international surveillance networks [92]. The WHO GLASS has scaled up to 195 countries by 2024 [93]. In addition to GLASS, other regional programs, such as the European Antimicrobial Resistance Surveillance Network (EARS-Net) and the CDC National Healthcare Safety Network (NHSN) in the United States, have made considerable progress in real-time monitoring of AMR. CDDEP’s ResistanceMap platform offers open-access visualizations of resistance trends for more than 100 countries. However, limitations of coverage remain for these tools, particularly in LMICs’ settings. Novel molecular surveillance approaches have improved the detection and tracking of resistant strains between countries [94]. Artificial intelligence-based real-time surveillance systems have shown enhanced capacity for early warning for the emergence of resistance patterns [95] (Figure 3).

Human, animal, and environmental surveillance data are integrated into One Health approaches for resistance monitoring [96]. Advances in diagnostic methods have transformed detection and characterization of resistance [97]. Standardization of antimicrobial susceptibility testing methods has improved global data compatibility [98] (Figure 3).

Next-generation sequencing technologies have enabled rapid identification of resistance mechanisms and transmission patterns [99]. Whole-genome sequencing (WGS) has transformed pathogen surveillance through detailed strain typing, tracking of transmission, and detection of resistance genes. Incorporating WGS into national surveillance programs may improve response times. Moreover, point-of-care molecular diagnostics (e.g., Xpert Carba-R, BioFire panels) can detect resistance genes within 2 h, including in field settings. Quality control systems for laboratory testing have been enhanced, with new international standards implemented across regions [100]. Point-of-care diagnostics have evolved to provide rapid resistance detection in various healthcare settings [101].

### 8.1. Applications of Artificial Intelligence in AMR Surveillance

Computer vision-based video surveillance tools that make use of deep learning techniques to detect or predict AMR are being developed or deployed on various platforms. DeepARG: towards predicting antibiotic resistance genes from metagenomic sequences using deep learning. An AI-based tool called pathogen.watch, developed by the Centre for Genomic Pathogen Surveillance (https://pathogen.watch/), combines genomic surveillance with AI to find outbreaks of high-risk clones. IBM’s Watson for Drug Discovery and other natural language processing tools have been used to scour the biomedical literature for emerging resistance threats. Hospital-based systems (e.g., Epic’s sepsis and antibiogram prediction modules) also leverage AI to aid clinicians with early detection and the best antibiotic-prescribing decisions.

Big data analytics have transformed the interpretation of surveillance data [102]. Machine learning algorithms have improved prediction models for resistance emergence and spread [103]. Integration of electronic health records with surveillance systems has enhanced real-time monitoring capabilities [104]. Standardized reporting frameworks have facilitated better international data comparison and trend analysis [105]. New visualization tools have improved the communication of surveillance data to stakeholders [106]. But, while advances have been made, many challenges remain. From the perspective of the LMICs, standard reporting systems, trained personnel and reliable laboratory infrastructure are often absent. Data fragmentation and insufficient interoperability are still major roadblocks. For this to be recognized, there must be global investment in both digital health infrastructure and equitable access to diagnostic technologies to strengthen surveillance.

### 8.2. Implementation Challenges in Surveillance and Laboratory Systems

Despite encouraging developments, major challenges remain to the effective realization of global AMR surveillance. Surveillance data remain limited and therefore not confirmatory due to a lack of basic laboratory infrastructure, human resource training and standardization of testing protocols in most LMICs. Data interoperability continues to be a challenge because electronic health record systems are often fragmented and not built for tracking resistance in real time. Widespread use of such next-generation diagnostics and molecules in resource-poor settings is also deterred due to their high costs. Moreover, the lack of sustainable funding models and long-term policy commitment leads to fragmentation of effort. Addressing these challenges will need worldwide investment in digital health infrastructure, workforce development and equitable access to diagnostic innovations.

## 9. Control Strategies, Clinical Interventions, Policy and Regulatory Measures

Evidence-based antimicrobial stewardship programs have demonstrated significant success in optimizing antibiotic use [107]. Implementation of rapid diagnostic testing has shown promising results in reducing unnecessary antibiotic prescriptions [108]. In both US and UK hospitals, the impact of multiplex PCR platforms has been striking, resulting in high reductions in empirical broad-spectrum antibiotic use. The availability of GeneXpert has aided in timely MDR-TB treatment initiation and an increase in treatment correctness rate in India. Novel infection prevention protocols have effectively reduced healthcare-associated resistant infections [109]. Patient-specific treatment algorithms incorporating local resistance patterns have improved clinical outcomes [110]. Digital health interventions have enhanced prescriber adherence to antimicrobial guidelines [111]. International policy frameworks have strengthened controls on antimicrobial use in both human and veterinary medicine [112]. National action plans have shown varying degrees of success in controlling antimicrobial resistance [113]. Regulatory changes restricting over-the-counter antibiotic sales have demonstrated positive impacts in multiple countries [114]. In Thailand and Vietnam, reductions in community antibiotic use of 15–20% within 3 years were achieved through government prohibition of OTC antibiotic sales combined with public education campaigns. As with Europe, a decline in colistin-resistant *E. coli* in food animals was detectable by 2022 in response to the ban. Economic incentives for pharmaceutical companies have stimulated new antimicrobial development [115]. Implementation of One Health policies has improved coordination between human health, animal health, and environmental sectors [116]. Healthcare provider education programs have effectively modified prescribing behaviors [117]. Public awareness campaigns have shown success in reducing inappropriate antibiotic demand [118]. Community-based interventions have improved understanding of antimicrobial resistance [119]. Social media campaigns have effectively reached younger populations with resistance prevention messages [120]. Professional development programs have enhanced healthcare workers’ compliance with stewardship principles [121]. In the future, a mixture of behavioral interventions, financial incentives, and robust legal frameworks will need to be employed to maintain those gains. Addressing a multidisciplinary audience, the 2023 Global AMR Summit stressed the necessity for integrated national strategies, greater accountability and codifying AMR targets within universal health coverage initiatives. Effective interventions should be situationally specific, flexible, and evidence-based.

### Implementation Challenges in AMR Control Policies

While the number of national and international AMR action plans continues to grow, there are some continuing challenges to implementation. These are insufficient funding, lack of political will and weak governance structures—especially in low-resource settings. Antibiotic prescription regulations are frequently poorly enforced in many nations because of fragmented health systems or drug markets that do not rely on formal distribution. Other challenges include limitations on sharing data between the human and veterinary health sectors, poor public engagement and a lack of strong accountability frameworks. Addressing these challenges will not be easy—it will depend on strong intersectoral cooperation, sustained investment, and incorporating AMR objectives into national health and development agendas.

## 10. Emerging Technologies and Research Priorities

Novel therapeutic approaches using CRISPR technology show promise in targeting resistant bacteria [122]. Artificial intelligence applications are advancing drug discovery and resistance prediction capabilities [123]. Innovative delivery systems for antimicrobials are being developed to enhance efficacy and reduce resistance development [124]. Nanotechnology-based interventions are emerging as potential solutions for resistant infections [125]. Advanced diagnostic platforms using quantum sensing show potential for ultra-rapid resistance detection [126]. Global research initiatives are focusing on understanding resistance transmission dynamics across different ecosystems [127].

The WHO 2024 Global AMR Surveillance Report shows resistance to third-generation cephalosporins in *E. coli* is now greater than 60% in some areas of South Asia, and resistance to carbapenem in *Klebsiella pneumoniae* has exceeded >50% in a number of Eastern European countries. Additionally, a 2023 Global AMR R&D Hub analysis identified that global investments for antimicrobial innovation only account for less than 1% of total health R&D spending. Although WGS technology is advancing, less than 35% of WHO member states have integrated whole-genome sequencing (WGS) into their AMR surveillance frameworks. This mismatch between innovation and implementation underscores the need for funding reallocation, regulatory harmonization, and improved data-sharing systems to support emerging tools such as CRISPR-based diagnostics, machine learning-guided treatment algorithms, and rapid molecular panels. The development of alternative therapeutic approaches, including bacteriophage therapy and immunomodulation, is gaining momentum [128]. Studies on microbiome manipulation as a resistance prevention strategy are showing promising preliminary results [129]. Investigation of novel drug targets through structural biology approaches continues to expand [130]. Research into social and behavioral aspects of antimicrobial use is becoming increasingly prioritized [131]. Future policy frameworks must address the complex interactions between human health, animal health, and environmental factors [132]. Economic models for sustainable antimicrobial development require innovative approaches to incentivize pharmaceutical investment [133]. Implementation strategies for new technologies need to consider resource-limited settings [134]. Global coordination mechanisms require strengthening to address cross-border resistance challenges [135]. Integration of emerging technologies into existing healthcare systems presents significant implementation challenges [136]. While this review includes studies from all WHO regions, the majority of available data stems from high-income countries with well-established surveillance and reporting infrastructures. Low- and middle-income countries (LMICs), despite bearing a disproportionate burden of antimicrobial resistance, often lack comprehensive surveillance systems, resulting in underreporting and potential data gaps. This geographic skew may lead to an underestimation of resistance trends and hinder the full global understanding of AMR patterns.

## 11. Conclusions

AMR remains a significant global health and economic threat. This review has identified a number of areas of significant concern in the changing AMR landscape, including (1) alarming increases in resistance rates amongst priority pathogens; (2) widening geographic gradients of surveillance and stewardship capabilities; (3) major delays in the pace of new antimicrobials reaching the clinical market; (4) the rising economic cost to health systems and productivity; and (5) the urgent need for coordinated, stakeholder-led, evidence-based policy responses. Our synthesis shows that while there have been partial successes for surveillance improvements and stewardship interventions, important gaps remain, particularly pronounced for low-resource settings and emerging pathogens. In this context, a new era of AMR response has to harness emerging diagnostic technology, cross-sector collaboration through One Health approaches, and increased global investment in antimicrobial research and development. In the future, the AMR crisis requires (a) equitable access to diagnostics and antimicrobials; (b) expansion of global surveillance platforms with real-time data sharing; (c) financial incentives for pharmaceutical innovation; as well as (d) inclusive policies that prioritize vulnerable populations. This needs to be an international effort to preserve our ability to fight infection and maintain a resilient health system for future generations. In contrast with previous reviews that have been narrow in scope, focusing on only individual aspects of AMR, this synthesis represents a comprehensive, multidomain analysis bridging together surveillance data, patterns of resistance, economic modeling, and health policy. This review highlighted key patterns in resistance emergence and containment gaps, emphasizing the need for context-specific interventions and renewed investment in surveillance, diagnostics, and equitable access to antimicrobials. Moreover, the review identifies less-studied challenges in LMICs, outlines cross-sectoral responses, and provides future perspectives, including AI-based diagnostic tools, CRISPR-based interventions, and One Health-based governance frameworks. This wide but actionable remit is designed to frame research priorities and strategic decision-making globally and nationally.

## Figures and Tables

**Table 1:** Summary of key multidrug-resistant pathogens. Prevalence varies by country and setting. Based on WHO/CDC data.

Pathogen	Resistance Mechanism	Prevalence (%)	High-Burden Regions	Last-Line Treatments
MRSA	Altered PBP (mecA)	20–40%	Global (esp. USA, India)	Linezolid, Daptomycin
VRE	VanA/VanB gene clusters	5–15%	Europe, Americas	Linezolid, Tigecycline
CRE	KPC, NDM, OXA-48 enzymes	10–50%	Asia, Middle East, Europe	Colistin, Ceftazidime-avibactam
ESBL-producing *E. coli*/*K. pneumoniae*	CTX-M enzymes	25–50%	Africa, South Asia	Carbapenems
*P. aeruginosa*, *A. baumannii*	Efflux pumps, OMP loss	20–60%	Global (esp. ICUs)	Colistin, Cefiderocol
*Candida auris*	Multidrug resistance (azoles, amphotericin B)	Rising	All continents	Echinocandins

Studies from 2020 to 2024 report increasing concerns about daptomycin-resistant and linezolid-resistant strains, particularly in tertiary care settings [37].

## Abbreviations

The following abbreviations are used in this manuscript:

AMR	Antimicrobial Resistance
MDRO	Multidrug-Resistant Organism
MRSA	Methicillin-Resistant *Staphylococcus aureus*
CRE	Carbapenem-Resistant Enterobacteriaceae
ESBL	Extended-Spectrum β-Lactamase
VRE	Vancomycin-Resistant Enterococci
NDM	New Delhi Metallo-β-lactamase
MCR	Mobilized Colistin Resistance
WHO	World Health Organization
CDC	Centers for Disease Control and Prevention
ECDC	European Centre for Disease Prevention and Control
GLASS	Global Antimicrobial Resistance Surveillance System
ICU	Intensive Care Unit
NGS	Next-Generation Sequencing
EHR	Electronic Health Records
OTC	Over The Counter
AST	Antimicrobial Susceptibility Testing
CRISPR	Clustered Regularly Interspaced Short Palindromic Repeats
AI	Artificial Intelligence
MDPI	Multidisciplinary Digital Publishing Institute
DOAJ	Directory of Open Access Journals
TLA	Three-Letter Acronym
LD	Linear Dichroism
DALY	Disability-Adjusted Life Year
GDP	Gross Domestic Product
LMIC	Low- and Middle-Income Countries
USD	United States Dollar
